# Harsh parenting and preschool children’s screen time: the mediating role of parent–child relationships and the moderating effect of mindful parenting

**DOI:** 10.3389/fpsyg.2025.1467701

**Published:** 2025-09-09

**Authors:** Caili Zhang

**Affiliations:** Faculty of Education, Xuchang University, Xuchang, China

**Keywords:** harsh parenting, parent–child relationship, mindful parenting, screen time, preschoolers

## Abstract

**Introduction:**

Excessive screen time among preschoolers is a growing public health concern. This study examined associations between harsh parenting and children’s screen time, testing parent–child relationship quality as a mediator and mindful parenting as a moderator.

**Methods:**

A cross-sectional survey was administered to 482 parents from four kindergartens in China (*Mage* = 36.0 years, *SD* = 3.89). Parents completed validated scales assessing harsh parenting, parent–child relationship quality, mindful parenting, and children’s daily screen time. A moderated mediation model was tested.

**Results:**

Harsh parenting showed an indirect association with greater child screen time through poorer parent–child relationships. Mindful parenting moderated the link between harsh parenting and parent–child relationship quality, such that higher mindful parenting buffered the negative impact of harsh parenting, attenuating the indirect effect on screen time.

**Discussion:**

Findings indicate that strengthening parent–child relationship quality and fostering mindful parenting may mitigate the influence of harsh parenting on preschoolers’ screen use. Interventions promoting positive, mindful parenting practices could help reduce excessive screen time in young children.

## 1 Introduction

### 1.1 Harsh parenting and child screen time

In today’s digital age, electronic devices have become ubiquitous, providing children with unprecedented access to various forms of screen media. This increased accessibility has led to a significant rise in screen time among preschool-aged children (Radesky et al., 2020; Radesky et al., 2015; Lauricella et al., 2015; Csibi et al., 2021). Research shows that infants spend increasing amounts of time on screens as they grow, with significant jumps in screen time between 2–4 months, 4–7 months, and 7–11 months, which can disrupt their daily routines from an early age (Krogh et al., 2021). This trend continues for young children, with their screen use reaching concerning levels that can be considered problematic (Radesky et al., 2020; Csibi et al., 2021; Park and Park, 2021; Christakis, 2009). While these devices can offer educational and entertainment benefits, too much screen time for young kids can harm both their physical and mental well-being. Studies indicate that prolonged screen use is linked to problems like trouble sleeping, obesity, and language development delays (Wu et al., 2023; Valkenburg, 2022; Pagano et al., 2023; Domoff et al., 2019). Additionally, excessive screen time can hurt kids’ social abilities and emotional control, potentially leading to behavioral issues (Yigiter et al., 2023; Paakkari et al., 2021; Oh et al., 2021; Huang, 2022). These findings highlight the importance of managing screen time for young children to ensure their healthy development.

Harsh parenting emerges as a potential factor influencing child screen time. Harsh parenting is characterized by coercive, punitive, and hostile behaviors towards children, including physical punishment, verbal aggression, and psychological control (Wang, 2017; Chen and Raine, 2018). Two theoretical perspectives help explain why harsh parenting might increase child screen time. First, the Stress-Coping Model (Lazarus, 1999) posits that individuals engage in various behaviors to manage stress. For children experiencing harsh parenting, excessive screen use may serve as a coping mechanism to alleviate emotional distress. Second, the Self-Determination Theory, which suggests that children have basic psychological needs for autonomy, competence, and relatedness (Deci and Ryan, 2000). Harsh parenting can thwart these needs, leading children to turn to screens as a means of gaining a sense of control and competence.

Research on adolescents has provided substantial evidence that harsh parenting can significantly impact their academic achievement (Wang et al., 2018), peer acceptance (Wang, 2017), social support (Lo et al., 2021), and life satisfaction (Ma and Song, 2023). Furthermore, harsh parenting practices have been consistently linked to the development of smartphone addiction, internet addiction, and short-form video addiction among adolescents (Lo et al., 2021; Yang et al., 2024; Wang and Qi, 2017; Wang et al., 2023; Wang et al., 2023; Wang et al., 2024; Lin et al., 2023). A common characteristic of these problematic media usage behaviors is the excessive amount of time spent on electronic media platforms. These findings suggest that harsh parenting practices may drive adolescents towards increased screen engagement, potentially as a coping mechanism for the stress and negativity present in their home environment. However, despite the well-established associations between harsh parenting and problematic screen use in adolescent populations, research examining these relationships in preschool-aged children remains markedly limited. This represents a significant gap in our understanding, particularly given that preschool years constitute a critical developmental period where early patterns of screen use are established and where intervention efforts could be most effective in preventing problematic behaviors before they become entrenched.

### 1.2 Parent–child relationship as the mediator

The impact of harsh parenting on child screen time may be mediated by the quality of the parent–child relationship. The Family Systems Theory provides a comprehensive framework for understanding this mediation. This theory posits that family members are interconnected, and the quality of interactions between parents and children can significantly influence children’s behaviors (Steinglass, 1987; Grusec, 2011). Harsh parenting can deteriorate the parent–child relationship, which in turn may lead to increased screen time as children seek alternative forms of engagement and comfort.

Harsh parenting negatively affects the parent–child relationship by creating an environment of fear and mistrust. Children subjected to harsh parenting often feel less secure and more anxious, which can strain their relationship with their parents (Wang and Qi, 2017; Wang et al., 2024). Research indicates that harsh parenting is associated with lower levels of parental warmth and responsiveness, leading to poorer attachment and increased conflict (Koehn and Kerns, 2018; Chung et al., 2022). The quality of the parent–child relationship can significantly influence child screen time. A positive parent–child relationship, characterized by warmth, responsiveness, and open communication, can reduce children’s reliance on screens by providing them with emotional support and meaningful interactions (Schneider et al., 2017; Detnakarintra et al., 2020). Conversely, a strained parent–child relationship can drive children towards screens as a means of escaping negative interactions and seeking comfort (Zhu et al., 2022; Sroufe, 2005; Shen et al., 2013).

In summary, the mediation pathway suggests that harsh parenting can lead to poorer parent–child relationships, which in turn can increase child screen time. This pathway is supported by both theoretical perspectives and empirical evidence, highlighting the importance of addressing the quality of the parent–child relationship in interventions aimed at reducing child screen time.

### 1.3 Mindful parenting as the moderator

Mindful parenting may moderate the relationship between harsh parenting and the parent–child relationship. Mindful parenting involves paying purposeful, present-moment, and non-judgmental attention to interactions with children (Kil et al., 2021; Bögels et al., 2010; Ahemaitijiang et al., 2021). It includes components such as listening with full attention, non-judgmental acceptance of self and child, self-regulation in the parenting relationship, emotional awareness of self and child, and compassion for self and child (Duncan et al., 2009). Mindful parenting can lead to improved parenting practices, better communication, and enhanced parent–child relationships, which in turn can result in positive outcomes for children, such as better emotional health and self-regulation, and decreased behavioral problems (Duncan et al., 2009; Jones, 2019; Han et al., 2021). Mindful parenting can help establish a positive parent–child relationship through two primary processes (Bögels et al., 2010; Ahemaitijiang et al., 2021). First, it can decrease parental stress, preoccupation, and the intergenerational transmission of dysfunctional parenting schemas and habits. Second, it can improve parental executive functioning, self-nourishing attention, marital functioning, and co-parenting. These improvements can create a more supportive and nurturing environment for children, which can mitigate the negative effects of harsh parenting.

In fact, empirical research has shown that mindful parenting can help alleviate perceived parenting stress, adjust parenting behaviors, and consequently foster positive daily parent–child interactions, leading to the maintenance of a healthy parent–child relationship (Coatsworth et al., 2018; Bögels et al., 2014; Anand et al., 2021; Passaquindici et al., 2024). These findings suggest that mindful parenting can buffer the negative impact of harsh parenting on the parent–child relationship, thereby reducing the likelihood of excessive child screen time.

### 1.4 The current study

Despite growing research on child screen time, the mechanisms through which parenting practices influence this behavior remain understudied, particularly for preschool children. Previous studies have often focused on direct effects, overlooking potential mediating and moderating factors. Additionally, the role of mindful parenting in the context of harsh parenting and child screen time has not been extensively explored.

**Figure 1:**
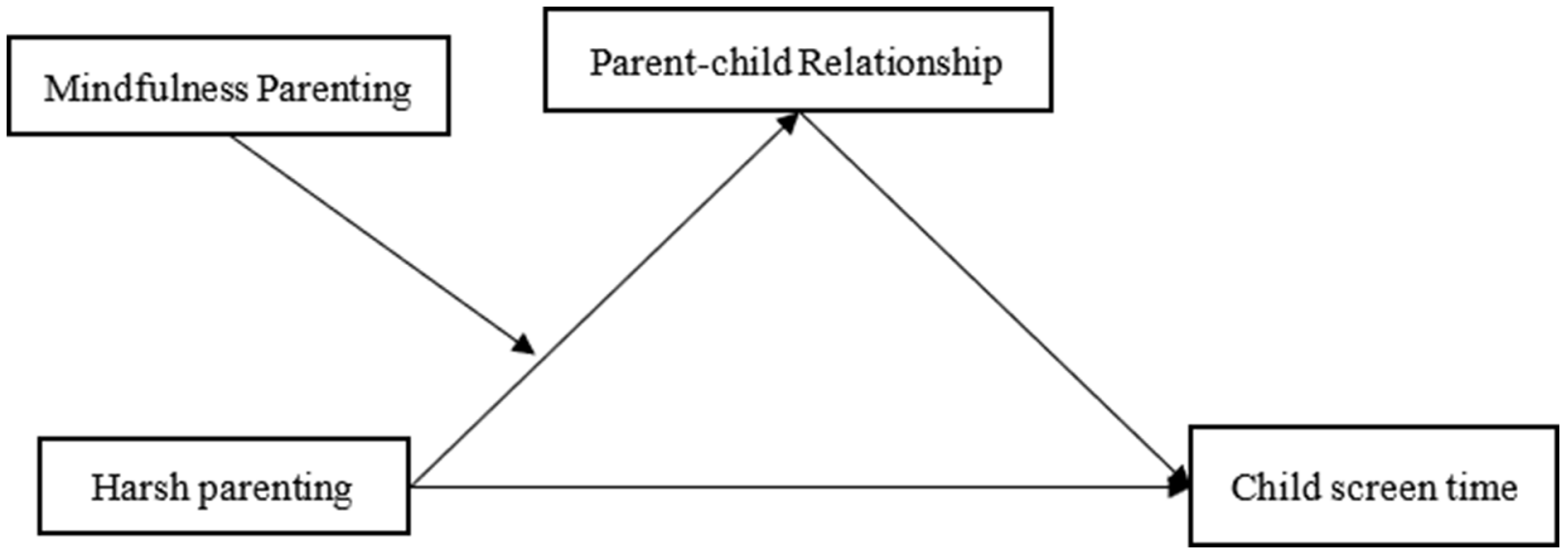
Diagram of the hypothesized moderated mediation model.

The current study aims to address these gaps by examining the relationship between harsh parenting and preschool child screen time, with a focus on the mediating role of parent–child relationship quality and the moderating effect of mindful parenting (see Figure 1). Based on the theoretical framework and empirical evidence reviewed above, we propose the following hypotheses:

Hypothesis 1: Harsh parenting will be significantly and positively associated with preschool children’s screen time. That is, parents who engage in more harsh parenting practices will have children with greater screen time usage.

Hypothesis 2: Parent–child relationship quality will mediate the relationship between harsh parenting and children’s screen time. Specifically, harsh parenting will be associated with poorer parent–child relationship quality, which in turn will be associated with increased child screen time.

Hypothesis 3: Mindful parenting will moderate the relationship between harsh parenting and parent–child relationship quality. As levels of mindful parenting increase, the negative association between harsh parenting and parent–child relationship quality will become progressively weaker.

## 2 Methods

### 2.1 Participants and procedure

This study surveyed parents from four kindergartens in Xuchang, Henan Province, China. Prior to the survey, the study received approval from the Ethics Review Committee of Xuchang University. The purpose of the survey was explained to the principals and teachers of the kindergartens, who then communicated the research information to the parents. Interested parents were invited to complete the questionnaire. As an incentive, parents received a small monetary reward upon completing the survey.

Prior to data collection, we conducted an *a priori* power analysis using G*Power 3.1 to determine the minimum required sample size for detecting the interaction term in our first-stage moderation (harsh parenting × mindful parenting) within a multiple regression framework. Assuming a small effect size for the interaction (*f^2^* = 0.02), *α* = 0.05, power (1 − *β*) = 0.80, and up to six predictors in the model (the two main effects, the interaction, and covariates), the analysis indicated a required sample of approximately *N* = 395.

The study initially approached 530 parents of preschool children through kindergartens. Of these, 48 participants were excluded due to incomplete questionnaire responses or poor response quality (e.g., selecting the same response option throughout the survey, indicating inattentive responding). This resulted in a final valid sample of 482 parents, yielding an effective response rate of 90.9%. The final sample comprised 482 parents with an average age of 36.0 years (*SD* = 3.89). Regarding educational attainment, 105 parents (21.8%) had a high school education or less, 133 (27.6%) had an associate degree, 212 (44.0%) had a bachelor’s degree, and 32 (6.6%) had a graduate degree or higher. The children in the study included 229 boys (47.5%) and 253 girls (52.5%), with an average age of 4.55 years (*SD* = 1.16). In terms of preschool grade level, the sample included 185 children (38.4%) in the senior class, 163 children (33.8%) in the middle class, and 134 children (27.8%) in the junior class.

### 2.2 Measures

#### 2.2.1 Harsh parenting

Harsh parenting was assessed using a four-item measure adapted from Wang and Qi (2017) for parent self-report. Parents were asked to evaluate how often they engaged in specific behaviors when their child did something wrong or made them angry. The four items were: “lose temper or even yell at the child,” “use an object to hit the child,” “hit the child with hands or feet,” and “tell the child to leave home.” Parents rated their own behavior on a five-point Likert scale ranging from 1 (never) to 5 (always). Higher scores indicated higher levels of harsh parenting. In this study, the Cronbach’s *α* for this scale was 0.912.

#### 2.2.2 Parent–child relationship

The Child–Parent Relationship Scale (CPRS), developed by Pianta and revised by Zhang et al. (2008), was employed to assess the quality of the mother–child relationship. The scale consists of 26 items, categorized into three dimensions: intimacy, conflict and dependency. Each item was rated on a 5-point Likert scale (1 = “not at all compliant,” 5 = “fully compliant”). For the purpose of this study, only the dimensions of intimacy and conflict were considered, as previous research has revealed low reliability within the dependency dimension (Zhang et al., 2008). The items in the conflict dimension were reverse-scored and then combined with the scores from the intimacy dimension. This scale demonstrated acceptable reliability (Cronbach’s α = 0.898 for intimacy, Cronbach’s α = 0.863 for conflict) in our study.

#### 2.2.3 Mindful parenting

Mindful parenting was assessed using the Chinese version of the Mindful Parenting Scale, revised by Pan et al. (2019). The scale includes 24 items across four dimensions: interacting with full attention, compassion and acceptance, self-regulation in parenting, and emotional awareness of the child. Items were rated on a five-point scale, from 1 (never true) to 5 (always true). The scale demonstrated good reliability and validity in this study (Cronbach’s α = 0.932.).

#### 2.2.4 Child screen time

Child screen time was measured using the method described by Domoff et al. (2019). Parents reported the time their children spent on eight types of screen media (TV, mobile phones, tablets, desktop computers, laptops, smart speakers with screens, smartwatches, and gaming consoles) on weekdays and weekends. The questionnaire categorized screen time into seven intervals: less than 15 min, 15–30 min, 31–60 min, 1–2 h, 2–3 h, 3–5 h, and more than 5 h. Midpoints of each interval were used, with more than 5 h assigned a value of 300 min. Average daily screen time was calculated as [(weekday screen time × 5) + (weekend screen time × 2)]/7.

### 2.3 Statistical analysis

Statistical analyses were conducted using SPSS 26.0. To address potential common method bias, Harman’s one-factor test was performed using principal component factor analysis. Descriptive statistics, including means, standard deviations, and Pearson correlations, were computed for all study variables. All variables were standardized prior to main analyses, with child gender, parental education, and parental age included as control variables throughout.

The hypothesized moderated mediation model was tested using Model 7 of the PROCESS macro for SPSS, which examines whether the indirect effect of harsh parenting on child screen time through parent–child relationship quality varies as a function of mindful parenting levels. Johnson-Neyman analysis was conducted to identify the specific range of mindful parenting values where harsh parenting significantly affects parent–child relationship quality. All analyses employed bootstrap resampling with 5,000 iterations to generate 95% bias-corrected confidence intervals, with effects considered significant when confidence intervals did not include zero.

## 3 Results

### 3.1 Common method bias

Given the use of questionnaires, common method bias was a potential concern. Harman’s one-factor test was conducted using principal component factor analysis (Podsakoff et al., 2003). Sixteen factors with eigenvalues greater than 1 were identified without rotation, with the first factor accounting for 19.26% of the variance, well below the 40% threshold. This indicated no significant common method bias.

It is acknowledged that Harman’s test is a relatively weak diagnostic for common method bias. However, the focal results rely on detecting specific moderated mediation patterns, including interaction effects and conditional indirect pathways. It is unlikely that a single common method factor could artificially generate such complex interaction and mediation effects, as method variance typically attenuates rather than creates interaction effects. Nevertheless, the single-informant design represents a limitation that future multi-informant studies could address.

### 3.2 Descriptive analysis

Table 1 presents the descriptive statistics and correlations for all variables. Harsh parenting was significantly negatively correlated with parent–child relationship and mindful parenting, and significantly positively correlated with child screen time. Parent–child relationship was significantly positively correlated with mindful parenting and negatively correlated with child screen time. No significant correlation was found between mindful parenting and child screen time.

**Table 1:** Descriptive statistics and correlations between variables (*n* = 482).

Variable	*M* ± *SD*	1	2	3	4	5	6
1 Child gender	−	1					
2 Parental education	−	−0.008	1				
3 Parental age	36.01 ± 3.90	−0.072	0.043	1			
4 Harsh parenting	2.02 ± 0.66	−0.044	−0.050	−0.043	1		
5 Parent–child relationships	3.45 ± 0.58	0.024	−0.006	−0.038	−0.378***	1	
6 Mindful parenting	3.61 ± 0.54	0.052	0.068	−0.059	−0.314***	0.599^***^	1
7 Child screen time	74.11 ± 54.54	−0.069	−0.026	−0.023	0.114*	−0.135^**^	−0.062

Child gender: boys = 0, girls = 1; parental education: 0 = high school and below, 1 = junior college education, 2 = college education, 3 = graduate education or above; ^*^p < 0.05, ^**^p < 0.01, ^***^p < 0.001.

### 3.3 Moderated mediation analysis

**Figure 2:**
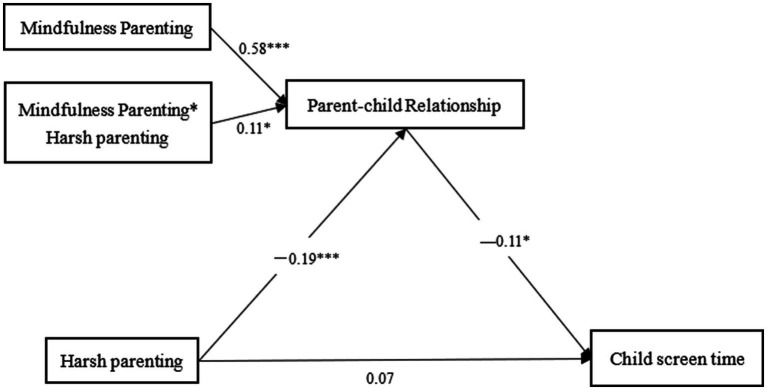
Results of moderated mediation analysis.

Model 7 of the PROCESS macro was used to test the moderated mediation model. As shown in Table 2, after including control variables, harsh parenting was significantly negatively related to parent–child relationship, and parent–child relationship was significantly negatively related to child screen time. There was no significant direct relationship between harsh parenting and child screen time, indicating that parent–child relationship mediated the effect of harsh parenting on child screen time. Additionally, the interaction between harsh parenting and mindful parenting was significantly related to parent–child relationship, suggesting that mindful parenting moderated the relationship between harsh parenting and parent–child relationship. The overall model is illustrated in Figure 2.

**Table 2:** Testing the moderated mediation effect of harsh parenting on child media use.

Predictors	Model 1 (Parent–child relationship)			Model 2 (Child screen time)		
	*β*	*t*	95% CI	*β*	*t*	95% CI
Child gender	−0.01	−0.14	(−0.149, 0.130)	−0.13	−1.45	(−0.310, 0.075)
Parent education	−0.06	−1.59	(−0.140, 0.015)	−0.03	−0.49	(−0.125, 0.075)
Parent age	−0.01	−0.25	(−0.202, 0.016)	−0.01	−0.62	(−0.030, 0.016)
Harsh parenting	−0.19	−4.88***	(−0.262, −0.112)	0.068	1.38	(−0.029, 0.164)
Parent–child relationship				−0.109	−2.23*	(−0.205, −0.013)
Mindful parenting	0.58	14.38***	(0.504, 0.663)			
Mindful parenting * Harsh parenting	0.11	3.00*	(0.038, 0.182)			
R2	0.413			0.168		
*F*	55.654***			2.765*		

Child gender, Parental age, and Parental Education are covariates. Child gender: boys = 0, Girls = 1. Mother education: 0 = high school and below, 1 = junior college education, 2 = college education, 3 = graduate education or above. ^*^p < 0.05, ^**^p < 0.01, ^***^p < 0.001.

**Figure 3:**
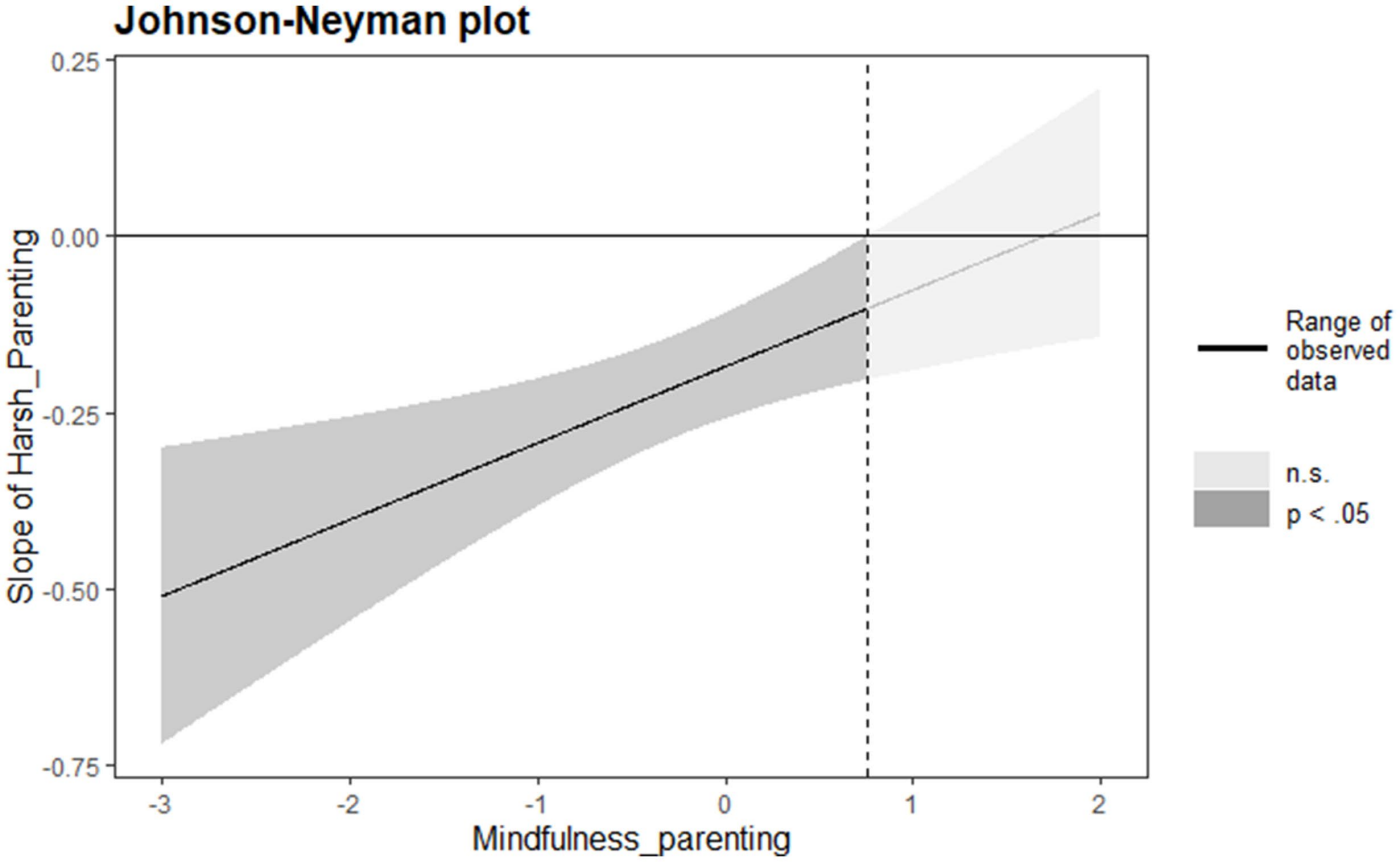
Johnson-Neyman plot demonstrates the moderating effect of mindful parenting on the relationship between harsh parenting and parent–child relationship. The *Y*-axis shows the regression coefficient of harsh parenting on parent–child relationship, while the *X*-axis represents the values of mindfulness parenting. In this study, mindfulness parenting range from −2.89 to 2.56. The bold solid line represents the simple regression coefficient, illustrating how harsh parenting and parent–child relationship vary with different levels of mindfulness parenting. The shaded area corresponds to the 95% confidence interval, indicating the division of statistical significance. The significant region is shown in black, while the non-significant region is in gray. The vertical dashed line represents the boundary of the significant region.

To further explore how mindful parenting moderates the relationship between harsh parenting and parent–child relationship, a Johnson-Neyman plot was used to illustrate this moderating effect. As shown in Figure 3, the relationship between harsh parenting and parent–child relationship varies depending on the level of mindful parenting. The Johnson-Neyman analysis revealed that when mindful parenting scores fall below 0.76 (within the observed range of −2.89 to 2.56), harsh parenting has a significant negative effect on parent–child relationship. However, when mindful parenting scores exceed 0.76, the negative effect of harsh parenting on parent–child relationship becomes non-significant. This finding suggests that higher levels of mindful parenting can effectively buffer against the detrimental effects of harsh parenting on parent–child relationship. Notably, the moderating effect of mindful parenting demonstrates a clear pattern: as mindful parenting increases, the negative impact of harsh parenting on parent–child relationship gradually weakens, as indicated by the upward slope in the Johnson-Neyman plot.

Mindful parenting also moderated the mediation effect of harsh parenting on child screen time through parent–child relationship, as shown in Table 3. When mindful parenting was low (M – 1 SD), the mediation effect was 0.032; when mindful parenting was high (M + 1 SD), the mediation effect was 0.008. Overall, higher levels of mindful parenting reduced the impact of harsh parenting on child screen time through the parent–child relationship.

**Table 3:** Analysis of the moderating effect of mindful parenting.

Indirect effect	Mindful parenting	Effect value	Boot SE	Boot LLCI	Boot ULCI
Indirect effect of harsh parenting on child screen time	M – 1 SD	0.032	0.016	0.004	0.067
	*M*	0.020	0.011	0.003	0.044
	M + 1 SD	0.008	0.008	−0.004	0.027

## 4 Discussion

### 4.1 Key findings

Firstly, this study found that harsh parenting negatively impacts child screen time through the mediating role of the parent–child relationship. This finding aligns with existing literature that highlights the detrimental effects of harsh parenting on various child outcomes. For example, previous studies have shown that harsh parenting is associated with increased behavioral problems and emotional distress in children (Chen and Raine, 2018; Wang et al., 2023; Lin et al., 2023; Liu et al., 2022). Our study extends this understanding by demonstrating that these negative outcomes can also manifest as increased screen time, a behavior often used by children as a coping mechanism to escape from stress and negative emotions induced by harsh parenting.

Theoretically, this finding can be explained through the lens of Family Systems Theory (Steinglass, 1987; Grusec, 2011), which posits that family members are interconnected, and changes in one part of the system affect the entire system. Harsh parenting behaviors disrupt the parent–child relationship, leading to a lack of emotional bonding and increased conflict. This difficult relationship might lead children to spend more time on screens as a way to escape the troubles and stress they are facing at home. The significance of this finding lies in its potential to inform targeted interventions. By highlighting the parent–child relationship as a key mediator, it suggests that interventions aimed at reducing child screen time should not only focus on direct screen management strategies but also on improving the overall quality of parent–child interactions and relationships.

Secondly, our study revealed that mindful parenting moderates the pathway from harsh parenting to the parent–child relationship. Specifically, as levels of mindful parenting increase, the negative impact of harsh parenting on the parent–child relationship diminishes. This finding is consistent with previous research demonstrating the positive effects of mindfulness in parenting contexts (Bögels et al., 2010; Ahemaitijiang et al., 2021; Jones, 2019; Han et al., 2021), but it provides a novel perspective on how mindful parenting can specifically buffer against the detrimental effects of harsh parenting practices.

To elucidate this moderation effect, we can turn to theoretical model of mindful parenting effects (Ahemaitijiang et al., 2021). This model posits that mindful parenting operates through two primary processes: individual internal processes and interpersonal processes. The individual internal processes, encompassing general mindfulness practices, likely enhance parental self-regulation and stress management. This improved self-regulation may help parents to respond more thoughtfully and less reactively, even in situations where they might otherwise resort to harsh parenting behaviors. The interpersonal processes, including awareness of the child, compassion, reduced automatic reactions, and non-judgmental acceptance of parent–child interactions, directly influence the quality of parent–child communication and relationships. These mindful approaches to parenting may create a more positive emotional climate within the family, potentially mitigating the negative impacts of occasional harsh parenting behaviors. Moreover, the buffering effect of mindful parenting on the harsh parenting-relationship pathway indirectly influences child screen time. By preserving the quality of the parent–child relationship, mindful parenting may maintain the parent’s ability to guide and regulate their child’s screen use effectively, even in the presence of some harsh parenting behaviors.

This finding is particularly important as it suggests a potential protective factor that could be cultivated to enhance family functioning and child outcomes. It offers hope that even in families where harsh parenting occurs, the cultivation of mindful parenting practices could help to maintain positive parent–child relationships and, by extension, promote healthier screen time habits in children. The implications of these findings are far-reaching. They not only contribute to our theoretical understanding of the complex dynamics within families but also offer practical insights for developing more effective interventions. By highlighting both the risks associated with harsh parenting and the protective potential of mindful parenting, this research paves the way for more nuanced and targeted approaches to promoting healthy child development in the digital age.

### 4.2 Implications

The findings of this study have several important implications for parents, educators, and policymakers concerned with children’s well-being in the digital era.

Firstly, the mediating role of the parent–child relationship in the association between harsh parenting and child screen time underscores the importance of fostering positive family dynamics. Parents should be made aware that their parenting style not only directly affects their children but also indirectly influences behaviors such as screen time through the quality of their relationship. This knowledge can motivate parents to reflect on their parenting practices and strive to create a more nurturing family environment. Parenting programs and interventions should emphasize the importance of building strong, positive relationships with children as a strategy for managing screen time, rather than focusing solely on restrictive measures.

Secondly, the moderating effect of mindful parenting offers a promising avenue for intervention. The finding that mindful parenting can buffer against the negative effects of harsh parenting on the parent–child relationship suggests that promoting mindfulness in parenting could be an effective strategy for improving family functioning and, consequently, children’s screen time habits. This has several practical implications: (a) Mindfulness-based parenting interventions should be developed and made widely accessible. These programs could teach parents skills such as present-moment awareness, non-judgmental acceptance, and emotional regulation, which are core components of mindful parenting. (b) Existing parenting education programs should consider incorporating elements of mindful parenting. This could help parents develop more adaptive responses to challenging situations, potentially reducing instances of harsh parenting and improving overall family dynamics. (c) Healthcare providers, including pediatricians and family doctors, could be trained to recognize the importance of mindful parenting and to provide basic guidance or referrals to appropriate resources.

Thirdly, parents should be encouraged to actively monitor their children’s screen time and be aware of the potential negative impacts of excessive screen use. Educational campaigns can provide parents with guidelines on recommended screen time limits and offer practical tips for managing screen use in the household. Parents can also be encouraged to engage in screen-free activities with their children, such as outdoor play, reading, and family games, to promote healthier alternatives to screen-based entertainment.

Fourthly, the individual internal process of mindful parenting, as highlighted in mindful parenting’s effects model, emphasizes the importance of parental health and well-being. Programs aimed at reducing child screen time should also address parental mental health and stress management. Providing parents with resources and support for managing their own stress and mental health can have a positive ripple effect on their parenting practices and the parent–child relationship. This holistic approach ensures that parents are better equipped to create a supportive and nurturing environment for their children.

In conclusion, the implications of this study extend beyond individual families to encompass broader societal approaches to supporting child development in the digital age. By addressing the root causes of excessive screen time through improving family dynamics and promoting mindful parenting, we may be able to create more sustainable and effective solutions to this pressing issue.

### 4.3 Limitations and future directions

While this study provides valuable insights into the relationships between parenting practices, parent–child relationships, and child screen time, several methodological limitations should be acknowledged, which also point to directions for future research.

Firstly, the cross-sectional nature of our data limits our ability to establish causal relationships between the variables studied. While our findings suggest that harsh parenting influences child screen time through the parent–child relationship, and that mindful parenting moderates this pathway, we cannot definitively conclude causality. Future research should employ longitudinal designs to track these relationships over time, allowing for more robust causal inferences. Such studies could also explore potential bidirectional relationships, considering how child screen time might, in turn, influence parenting practices and parent–child relationships.

Secondly, the reliance on self-report measures may have introduced common method bias and social desirability bias. Parents’ perceptions of their own parenting practices, their relationship with their child, and their child’s screen time may not always accurately reflect reality. More specifically, social desirability bias may have led parents to under-report harsh parenting behaviors (which are generally viewed as socially unacceptable) and over-report mindful parenting behaviors (which are perceived as socially desirable). This systematic bias in reporting could potentially attenuate the observed effects, as the true range of harsh parenting behaviors may be underestimated while mindful parenting behaviors may be overestimated. Such response biases would likely reduce the statistical power to detect relationships and may mean that the actual associations between parenting practices and child screen time are stronger than those observed in this study. Future studies should consider incorporating multiple methods of data collection, such as observational measures of parent–child interactions, objective measures of screen time (e.g., device usage logs), and reports from multiple informants (e.g., teachers, children themselves when age-appropriate). This multi-method approach would provide a more comprehensive and potentially more accurate picture of family dynamics and child behaviors.

Thirdly, our sample was drawn from a specific geographic region and may not be representative of broader populations with different cultural, socioeconomic, or demographic characteristics. The generalizability of our findings across diverse cultural contexts, varying socioeconomic backgrounds, and different family structures remains to be established. Future research should examine these relationships in more diverse samples to enhance external validity.

Additionally, future studies could explore other potential moderators or mediators in the relationship between parenting practices and child screen time. Factors such as parental screen time habits, family media rules, or children’s individual characteristics (e.g., temperament) could play important roles in these relationships.

Addressing these limitations in future research will contribute to a more nuanced and comprehensive understanding of the complex interplay between parenting practices, family relationships, and children’s screen time behaviors. This knowledge will be crucial for developing more effective interventions and policies to promote healthy digital media use among children.

## 5 Conclusion

This study provides valuable insights into the mechanisms through which parenting practices influence child screen time. The findings highlight that harsh parenting indirectly increases child screen time by deteriorating the quality of the parent–child relationship. Importantly, mindful parenting can buffer these negative effects, suggesting that promoting mindfulness in parenting can improve family dynamics and reduce children’s reliance on screens. These results underscore the need for interventions that focus on enhancing parent–child relationships and incorporating mindful parenting practices. By addressing these underlying factors, we can develop more effective strategies to manage screen time and promote healthy development in young children.

## Data Availability

The raw data supporting the conclusions of this article will be made available by the authors, without undue reservation.
